# Clinical results of femoral head fracture-dislocation treated according to the Pipkin classification

**DOI:** 10.12669/pjms.333.12633

**Published:** 2017

**Authors:** Xiao Yu, Qing-Jiang Pang, Xian-Jun Chen

**Affiliations:** 1 Dr. Xiao Yu, PhD. Department of Orthopedics, Ningbo No.2 Hospital, Ningbo, 315010, Zhejiang, China; 2 Dr. Qing-Jiang Pang, PhD. Department of Orthopedics, Ningbo No.2 Hospital, Ningbo, 315010, Zhejiang, China; 3 Dr. Xian-Jun Chen, MD. Department of Orthopedics, Ningbo No.2 Hospital, Ningbo, 315010, Zhejiang, China

**Keywords:** Femoral head fracture-dislocation, Harris Hip Score’s criteria, Pipkin classification

## Abstract

**Objective:**

To summarize the clinical results of femoral head fracture-dislocation treated according to Pipkin classification.

**Methods:**

Atotal of 19 patients with femoral head fracture-dislocation were retrospectively analyzed from Mar. 2008 to Mar. 2015. According to the classification of Pipkin criteria, there were 4 cases in Type-I, 6 cases in Type-II, 6 cases in Type-IIIand 3 cases in Type-IV. Various procedures were taken according to the different types of the fracture, the time of the fracture, and the age of the patients. X-ray was examined during the follow-up period and functional evaluation was carried out by Harris Hip Score’s criteria. The clinical therapeutic effects were analyzed.

**Results:**

All the patientsgot a mean follow-up of 18 months (9-36 months). No patient suffered from infection, skin flap necrosis and X-ray showed no implants loosening or breakage. According to the Harris Hip Score’s criteria, in Type-I, 4 cases were rated as excellent. In Type-II, 2 cases rated as excellent, 3 cases as good and 1 case as fair. In Type-III, 3 cases rated as good, 2 cases as fair and 1 case as poor. In Type-IV, 1 case rated as excellent, 1 case as good and 1 case as fair. The overall rate of excellent and good was 73.7%.

**Conclusions:**

Pipkin classification is helpful to make preoperative plan and judging the prognosis in cases of femoral head fracture-dislocation. However, multiple factors such as the time from injury to surgery, the ages of patients, the selection of implants should also be considered, which may affect the clinical results.

## INTRODUCTION

Femoral head fracture-dislocation, alternatively also called Pipkin fracture, is mostly caused by high energy injuries.^1^ Because it is seldom seen in clinical practice, the surgeons always focus on the femoral head dislocation and the minor fracture fragments from the femoral head can be easily missed.^2^ The missed diagnosis of Pipkin fracture will cause poor prognosis unless the patient accepts the total hip arthroplasty (THA) on initial presentation since it can be easily cause late onset of femoral head necrosis and traumatic arthritis.^3^ However, for the young patients, offering a primary replacement arthroplasty for traumatic fracture-dislocation maybe controversial. Therefore, the therapeutic requirements and difficulties of Pipkin fracture are high.

At present, the Pipkin classification is widely used in the femoral head fracture-dislocation^4^,^5^ however, seldom has the literature linked a Pipkin fracture with preoperative plan and prognosis. In this study, we have retrospectively analyzed 19 patients’ clinical results of femoral head fracture-dislocation.

## METHODS

### Clinical data

A total of the 19 patients (15 males and 4 females) of femoral head fracture-dislocation were treated from Mar. 2008 to Mar. 2015. The age ranged from 25 to 63 years old (40.2years old in average). All the patients were diagnosed as unilateral femoral head injury. According to Pipkin classification, there were 4 cases in Type-I, 6 cases in Type-II, 6 cases in Type-III and 3 cases in Type-IV. Furthermore, 2 cases in Type-III were had a concomitant fracture of the superior and inferior ramus of the pubis on the same side. In one case, Type-IV had an injury of sciatic nerve.

### Treatment methods

Conservative therapy of femoral supracondylar traction reduction was performed on 3 cases of Type-Iand 1 case of Type-II. The other 15 cases were treated surgically. Pre-operative femoral supracondylar traction was carried out once the patients were hospitalized. Of all the 15 cases subjected to surgical treatment, the femoral head was reduced within 48 hours after the injury in 9 cases, 48 hours~1 week in 3 cases and 1 week~2 weeks in 3 cases. 1 case in Type-IV underwent emergent nerve exploration for the concomitant injury of the sciatic nerve. 3 cases in Type-IV underwent internal fixation by reconstruction plates for the acetabulum and absorbable screws for the femoral head respectively after the vital signs were stable. 1 case aged 63 in Type-III was treated with total hip arthroplasty, while the other 2 cases were treated with open reduction by cannulated screw fixation. The rest of the 3 cases with closed reduction by cannulated screwfixation leaving the femoral head fractures in non-weight bearing area untreated. The other cases, whose fracture belonged to Type-I and Type-II were treated with open reduction and absorbable screws fixation. In the cases of open reduction and internal fixation, the Smith-Peterson approach was used in 4 cases while the Kocher-Langenbeck approaches in 7 cases.

### Postoperative management

Postoperatively, femoral supracondylar traction was used to the cases treated with open or closed reduction and internal fixation for another 8 weeks. The patients were encouraged to do the quadriceps femoris rehabilitation exercise when as pain decreased.^6^ However, the patient with THA was asked to walk full weight-bearing within 2 weeks postoperatively. X-ray examination was carried out once a month during the first 3 months then it could be taken at the 6^th^ month and the 12^th^ month to evaluate the efficacy of reduction maintenance and judge whether the femoral head necrosis occurred. Regular follow-up was maintained and the functional recovery was evaluated according to the Harris Hip Score’s criteria when the patient can walk full weight-bearing.

## RESULTS

**Fig.1:**
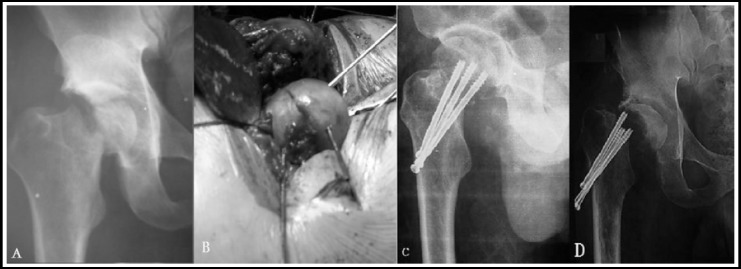
A male patient, with Type-III Pipkin fracture (A), In the initial surgery, the femoral head ligament was cut to reduce the femoral head fracture with cannulated screws. (B, C), In the 36^th^ month follow-up, he suffered from pain and joint movement disorder with obvious collapse and necrosis of the femoral head showed on X-ray. The Harris Hip Score was rated as poor (D).

All the 19 patients were accepted followed-up for 9-36 months (18 months in average) and the postoperative complications such as infection, skin flap necrosis, loosening or breakage of the implants, nounion or malunion were not detected. In every visit, pain, gait, range of the motion, weight-bearing of the joint were evaluated and the X-ray were taken. A note was made of joint space of the hip, the collapse of the femoral head, and the heterotopic ossification of the hip joint. According to Harris Hip Score’s criteria, 4 case in Pipkin Type-I were rated as excellent; in Type-II, 2 cases were rated as excellent, 3 as good, and 2 as fair; in Type-III, 3 as good, 2 as fair and 1 as poor; and in Type-IV 1 as excellent, 1 as good and 1 as fair, the overall rate of excellent and good was 73.7%. The case scoring as poor in Type-III suffered from pain, with obvious collapse and necrosis of the femoral head showed on his X-ray in his 36^th^ month follow-up (Fig.1).

## DISCUSSION

The types of Pipkin fracture depends on location and direction of force from femoral head to acetabulum. If flexion degree of hip joint is >60° in the moment of trauma, Type I or II Pipkin fracture could occur. However, if flexion degree is <60°, it could result in Type III or IV fracture.^7^ Pipkin classification is significant in making preoperative plan and predicting prognosis.

The femoral head necrosis occurs due to lack of blood supply resulted from prolonged dislocation of the hip joint and accelerates if the femoral head ligament is cut in operation to deteriorate the blood supply.^8^ Traumatic arthritis may develop due to poor reduction of fracture in the weight bearing area of the articular surface. Therefore, the management aim is to restore the articular surface as soon as possible.^6^ In this study, 9 cases was scheduled to operation within 48 hours. 2 cases of Type-III concomitant the fracture of the superior and inferior ramus of the pubis underwent operation 48 hours to a week. 3 cases of Type-IV were treated a week later for their seriously concomitant fracture of acetabulum. The results showed the cases treated earlier than 48 hours showed better outcome since the remaining blood supply of the femoral head may be better preserved if the reduction can be achieved early.

We achieved a satisfying overall result in 19 patients in their follow-up as mean rate of excellent and good in four types was 73.7%, especially in 3 cases of Type-IV. Therapeutic results were related to time of follow-up for femoral head necrosis and traumatic arthritis may not appear in a short time. In this study, the case accepted the operation a week later showed satisfying result according to the latest follow-up. We believed it was attributed to the femoral supracondylar traction, which was helpful to reduce the swelling of the soft tissue in the hip joint.

Choice of approach is controversial.^9^,^10^ The approaches often used are anterior approach Smith-Peterson and posterior approach Kocher-Langenbeck. The advocates of the posterior approach believe that most femoral head dislocation fall into the category of the posterior dislocation, in which the simultaneous posterior capsular lancinate usually occurred and the blood supply for the femoral head from the medial femoral circumflex artery would be destroyed. Therefore, if the anterior approach was used, it would destroy the left blood supply of femoral head because the ascending branch of lateral femoral circumflex artery and anterior capsular must be cut by this approach.^11^ While some scholars believe that the posterior approach is difficult to expose, reduce and fix the fractures in the anterior, medial and inferior parts of femoral head unless the incision became longer.^12^ It is advisable to choose anterior approach to expose, reduce and fix for Type-I or Type-II as the fracture was in the anterior and inferior of the femoral head. While for Type-III or Type-IV, the posterior approach is more convenient for reduction and fixation of posterior wall fracture of acetabulum.^13^

Anatomic reduction and effective fixation of femoral head fracture can reduce the traumatic arthritis rate. It is advocated to surgical management as long as it is indicated. As for Type-I, the fracture could be removed if it is small in non-weight bearing area. However, as for fracture larger than 1/3 of joint surface, it should be preserved and treated with internal fixation. Open reduction should be done as early as possible in Type-II since the fracture is always large in weight bearing area.^14^ The implants can be divided into metal screw and absorbable screw. The scholars who preferred absorbable screw believe that strength of absorbable screw is enough and the stress would be transferred to healing bone surface.^15^ In this study, 11 cases were fixed by absorbable screws and showed satisfying clinical results. As for the cases of Type-III, the rate of femoral head necrosis will increase enormously for the destruction of blood supply of the femoral head by open reduction. Therefore, we believe that the indication for THA should be extensive in the cases of Type-III. In this study, the patient in Type-III were treated by THA and showed no claudication and pain in 12^th^ month follow-up. As the fracture in Type-IVconcomitant acetabulum fracture, it is difficult to maintain the state of reduction after traction, and cartilage degeneration and joint stiffness may occur if joint is fixed for a long time. Furthermore, the fracture of acetabulum should be fixed simultaneously with the reduction of femoral head in order to prevent malalignment between acetabulum and femoral head.^16^

The rate of sciatic nerve injury caused by Pipkin fracture was about 10% and approximately, 60%-70% of the sciatic nerve injury could recover.^6^ In this study, 1 case accepted emergency nerve exploration for concomitant injury of sciatic nerve and we found that the femoral head in posterior dislocation lancinated the posterior capsular. Therefore, the sciatic nerve was in the state of distraction and entrapment. The rate of traumatic arthritis in femoral head fracture-dislocation is 24% and it will increase when concomitant acetabulum fracture, unanatomical reduction and delayed reduction.^8^ The femoral head necrosis may be related to time from injury to surgery, original or iatrogenic injury of medial femoral circumflex artery. We believed that in order to avoid the femoral head necrosis in a maximum degree, the reduction operation should be taken as early as possible. In this study, 1 case of Type-III suffered from Pipkin fracture concomitant superior and inferior ramus of the pubis accepted open reduction and internal fixation after 48 hour later when the active bleeding was excluded. However, in the process of operation, the ligament of femoral head was cut to take out of the fracture bone then it was fixed to the femoral head with cannulated screw. Therefore, the blood supply of the femoral head was destroyed seriously. In the 36^th^ month follow-up, pain and joint movement disorder with obvious collapse and necrosis of the femoral head appeared. Harris hip score was rated as poor.
